# A Novel Grain-Based DEM Model for Evaluating Surface Integrity in Scratching of RB-SiC Ceramics

**DOI:** 10.3390/ma15238486

**Published:** 2022-11-28

**Authors:** Huan Qi, Yuelei Wang, Zijian Qi, Liwu Shi, Zhufang Fang, Li Zhang, Oltmann Riemer, Bernhard Karpuschewski

**Affiliations:** 1 College of Mechanical Engineering, Zhejiang University of Technology, Hangzhou 310023, China; 2 Department of Manufacturing Technologies, Leibniz Institute for Materials Engineering-IWT, 28359 Bremen, Germany; 3 MAPEX Center for Materials and Processes, University of Bremen, 28359 Bremen, Germany; 4 State Key Laboratory of High Performance Complex Manufacturing, Central South University, Changsha 410083, China; 5 School of Energy Science and Engineering, Central South University, Changsha 410083, China

**Keywords:** grain-based DEM model, cluster, RB-SiC ceramics, scratching process, surface integrity

## Abstract

A novel grain-based DEM (Discrete Element Method) model is developed and calibrated to simulate RB-SiC (Reaction-Bonded Silicon Carbide) ceramic and associated scratching process by considering the bonded SiC and Si grains and cementitious materials. It is shown that the grain-based DEM model can accurately identify transgranular and intergranular cracks, and ductile and brittle material removal modes. It also shows that by increasing the scratching speed or decreasing the depth of cut, the maximum depth of subsurface damage decreases, because the scratching force is relatively large under the low scratching speed or large depth of cut that facilitates the occurrence of transgranular cracks, large grain spalling from the target surface and the propagation of median cracks into the target subsurface. It has further been found that increasing the cutting-edge radius can enhance the target ductile machinability and reduce the target subsurface damage.

## 1. Introduction

Ceramics have excellent physical and chemical properties, finding applications in aerospace, electronics, military, etc. [1,2,3]. The RB-SiC (Reaction-Bonded Silicon Carbide) ceramics, in particular, have become an attractive avenue for investigation by many researchers, due to its increasing demand for use in molding dies, chip substrates and information technology, whose performances greatly depend on a surface integrity with no or minimum process-induced surface/subsurface damage by cutting, grinding and polishing processes [4,5]. Hence, an understanding the material removal mechanisms of RB-SiC ceramics is essential to obtain a good surface finish and facilitate their practical application.

**Figure 1:**
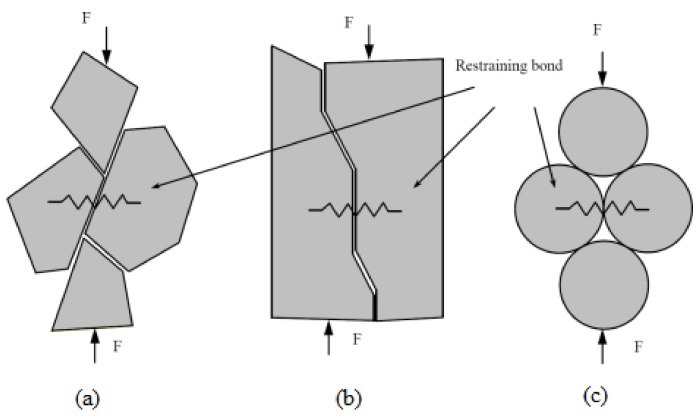
Comparison of the formation of cracks between a real target and the solid specimen constructed with the Bonded Particle Model: (**a**) physical crack between grains, (**b**) physical crack inside grains, and (**c**) breakage (crack) among spherical elements in a solid specimen [13].

Abrasive machining technology as a non-traditional machining technology has been extensively employed to polish a variety of materials, especially the hard-to-machine materials. Chen et al. [6] proposed a novel shear dilatancy polishing method which was employed to precision-polish tungsten surface, and it was found that not only had the material removal rate been significantly improved by about 33.4%, but a better surface finish could be obtained. By using this method, Chen et al. [7] also investigated the effect of the surface quality on hydrogen/helium irradiation behavior in tungsten, which indicated that improvement of the surface quality could essentially enhance the ability of the resistance to hydrogen/helium irradiation behavior in tungsten. Since abrasive machining technology is very useful in the precision machining area, the fundamentals of this technology should be investigated as well. Scratching technology with a cutter or single abrasive is widely used in exploring the fundamentals of material removal mechanisms in abrasive machining of hard brittle materials [8]. Shi et al. [9] investigated edge chipping in the scratching of soda-lime glass, and found that considering the high strain-rate hardening effect led to a more accurate prediction of edge chipping and subsurface damage, which could provide suggestions in the fabrication of high-value optical devices. Moreover, Huang et al. [10] explored the plastic deformation and crack propagation caused by the scratching of single crystal alumina, where the plastic deformation could be attributed to the coupling effects of lattice disorder, dislocation loops, stacking faults, dislocation glide and basal twin formation. For ceramics which are made of bonded crystal grains with some enhanced materials at given high temperatures, it was found that both intergranular and transgranular fractures occurred during the machining of aluminium nitride ceramics [11]. Liu et al. [12] explored the material removal mechanisms in the processing of alumina ceramics based on scratching tests, and found that transgranular fracture, consisting of ploughing and ductile cutting, dominated the material removal process. Although some fundamental material removal mechanisms could be concluded by analysing the morphology of eroded surfaces, the dynamic scratching process could not be observed experimentally due to the very short scratching period between the tool and target surface and very small cracks formation inside the target as illustrated in Figure 1.

Numerical simulation is alternatively used to investigate some dynamic and complicated problems that cannot be addressed experimentally [14,15,16,17]. DEM, treating the brittle materials as a solid specimen with arbitrarily sized spherical elements bonded together with the assistance of the BPM (Bonded Particle Model), can model mechanical behaviour subjected to external force or moment, which are mainly governed by the formation, propagation and eventual intersection of cracks [13]. Figure 1 shows a comparison of the formation of cracks between a solid specimen constructed with BPM and a real target material. Physical cracks between and inside grains in a real target material occur once a large enough force or moment is applied to the target material as shown in Figure 1a,b, respectively, while similar breakages (cracks) also occur among the spherical elements in a solid specimen, as shown in Figure 1c. Many such cracks would occur inside a solid specimen that contains thousands of bonded spherical elements to represent the cracking process in brittle materials, especially for RB-SiC ceramic which is made of bonded grains by the addition of enhanced materials at given temperatures. Jiang et al. [18,19,20] employed DEM to model SiC ceramics, and explored the crack propagation and failure mode by simulating uniaxial compression and three-point bending tests. Zhang et al. [21] modelled ultrasonic-assisted scratching of alumina ceramic using DEM, and found that the crack initiation was restricted by the addition of compressive stress on the target during the machining process. However, these studies did not consider the different grains in the ceramics, such as RB-SiC ceramic which is mainly made of bonded SiC and Si grains, and the propagation of cracks inside and along these grains, namely, the intergranular and transgranular fractures, during the machining process. Thus, a more accurate model considering the effect of the different grains inside RB-SiC ceramic on the material removal process has been developed in this paper to represent the scratching process more realistically.

Thus, in this paper, a novel grain-based DEM model was developed to construct a solid specimen of RB-SiC ceramic by considering the SiC grain, Si grain and cementitious materials, respectively, with the assistance of the cluster model and parallel bond model, which are extensively used to model hard-brittle materials with bonded structures, using Particle Flow Code 2D (PFC2D) version 5.0 software. Then, the macroscopic properties of the constructed solid specimen were numerically calibrated to agree well with the corresponding material properties of the real RB-SiC ceramic. Finally, the scratching of RB-SiC ceramic was numerically modelled by considering different processing parameters, and their effects on the surface integrity were also explored for providing an effective way to improve machining performance.

## 2. Methodology

### 2.1. The Grain-Based DEM Model of RB-SiC Ceramic

Since DEM is a promising approach to simulate the erosion process for brittle materials using the BPM, it is necessary to understand the underlying science of this model. The forces and moments between these spherical elements can be transmitted in the parallel bond model [22], and the contact force, ${\bar{F}}_{i}$, and moment, ${\bar{M}}_{i}$, carried by the parallel bond can be divided into normal and shear components that are given by:

(1) $${\bar{F}}_{i}={\bar{F}}_{i}^{n}+{\bar{F}}_{i}^{s}$$

(2) $${\bar{M}}_{i}={\bar{M}}_{i}^{n}+{\bar{M}}_{i}^{s}$$

Hence, the resultant maximum tensile stress, *σ*_max_, and shear stress, *τ*_max_, on the parallel bonds among the spherical elements can be expressed as:

(3) $$\sigma_{\max}=\frac{-{\bar{F}}^{n}}{A}+\frac{\left|{\bar{M}}^{s}\right|}{I}\bar{R}$$

(4) $$\tau_{\max}=\frac{\left|{\bar{F}}^{s}\right|}{A}+\frac{\left|{\bar{M}}^{n}\right|}{J}\bar{R}$$

where ${\bar{F}}^{n}$, ${\bar{F}}^{s}$, ${\bar{M}}^{n}$ and ${\bar{M}}^{s}$ are the scalar values of ${{\bar{F}}_{i}}^{n}$, ${{\bar{F}}_{i}}^{s}$, ${{\bar{M}}_{i}}^{n}$ and ${{\bar{M}}_{i}}^{s}$, respectively, $\bar{R}$ is the parallel bond radius, *A* is the area of the parallel bond cross-section, and *I* is the moment of inertia (for the 2D model, *J* is null).

It can be found from Equations (3) and (4) that in the normal direction, the bond breakage meets the maximum tensile stress criterion, while in the shear direction it meets the Mohr–Coulomb criterion, so that two failure modes, including tension failure and shear failure, occur if either of these maximum stresses exceeds its corresponding bond strength. In this way, it can be used to simulate the formation of cracks in brittle materials subject to the external force or moment, and hence, to explore the related material removal mechanisms.

**Figure 2:**
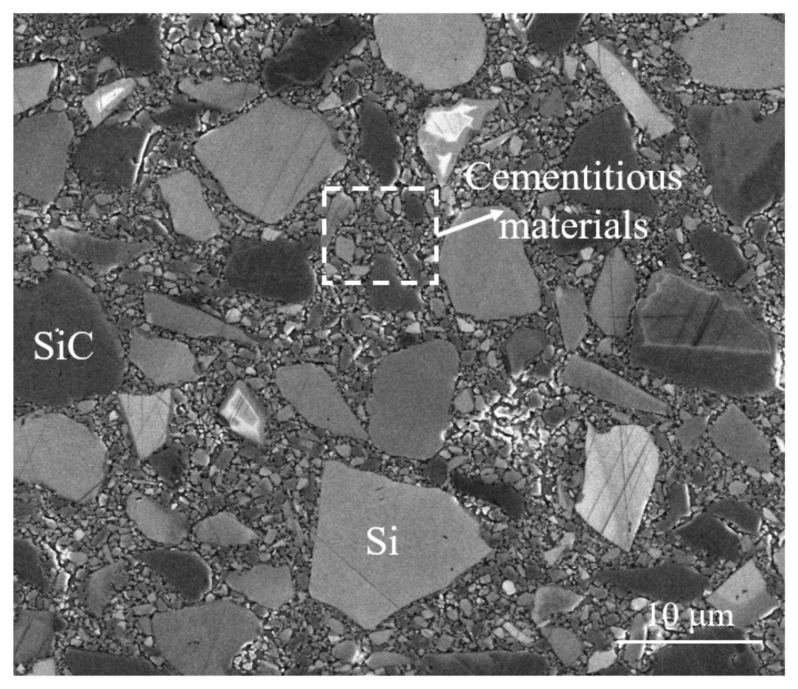
SEM image of the RB-SiC ceramic.

Furthermore, it is noted from Figure 2 that RB-SiC ceramic is mainly made of bonded SiC and Si grains, and also includes cementitious materials, being carbon powders, bonding agents and free SiC and Si grains. In general, the cracks between these bonded SiC and Si grains are considered as intergranular fractures, while the cracks inside these bonded SiC and Si grains are regarded as transgranular fractures, as can be observed from Figure 1a,b, respectively. Due to the different properties of SiC, Si and cementitious materials, both intergranular and transgranular fractures could occur in the scratching of RB-SiC ceramic which, in turn, could affect the surface/subsurface quality; hereby a novel grain-based DEM model is needed to accurately simulate RB-SiC ceramic as well as to evaluate the scratching process, as detailed below.

**Figure 3:**
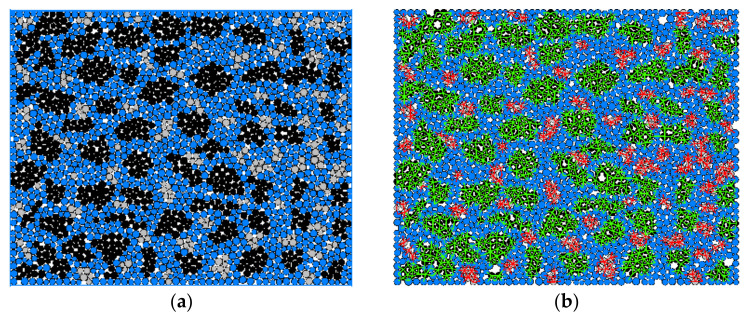
(**a**) A solid specimen with different clusters (black, grey and blue spherical elements represent the SiC grain, Si grain and cementitious materials, respectively), and, (**b**) the parallel bonds in different clusters (green lines represent the parallel bonds in SiC grains and red lines represent the parallel bonds in Si grains).

In PFC2D, a cluster model is usually employed to simulate the grain-based materials, and is defined as a set of spherical elements that are adjacent to one another, where each cluster is grown by identifying the current spherical element as a seed and then adding adjacent special elements to the cluster until the desired cluster size has been reached. Then, the different microscopic properties of the parallel bond can be added inside and between each cluster, so that if this kind of solid specimen is subjected to a large enough force or moment, these cracks may occur inside or along the boundary of clusters, which can simulate the intergranular and transgranular fractures of grain-based materials. Thus, a novel grain-based DEM model combining the cluster and parallel bond model is developed here to simulate the real RB-SiC ceramic that consists of bonded SiC and Si grains and cementitious materials as shown in Figure 3a, which is similar to the real RB-SiC ceramic. The associated parallel bonds in different clusters are given in Figure 3b, which should be calibrated by numerical tests, including the compression test, three-point bending test and single-edge notch bending test, relying on the corresponding physical test results, as detailed below.

### 2.2. Model Calibration

Since the major material properties, such as Poisson’s ratio, bending strength, Young’s modulus and fracture toughness, dominate the mechanical behaviours of RB-SiC ceramic in the material removal process during the machining process, it is first necessary to validate these macroscopic properties of the target solid specimen constructed in Section 2.1, and one of the most critical steps in PFC2D modelling is the calibration of input microscopic properties for the spherical element and parallel bond in different clusters, as given in Table 1.

**Figure 4:**
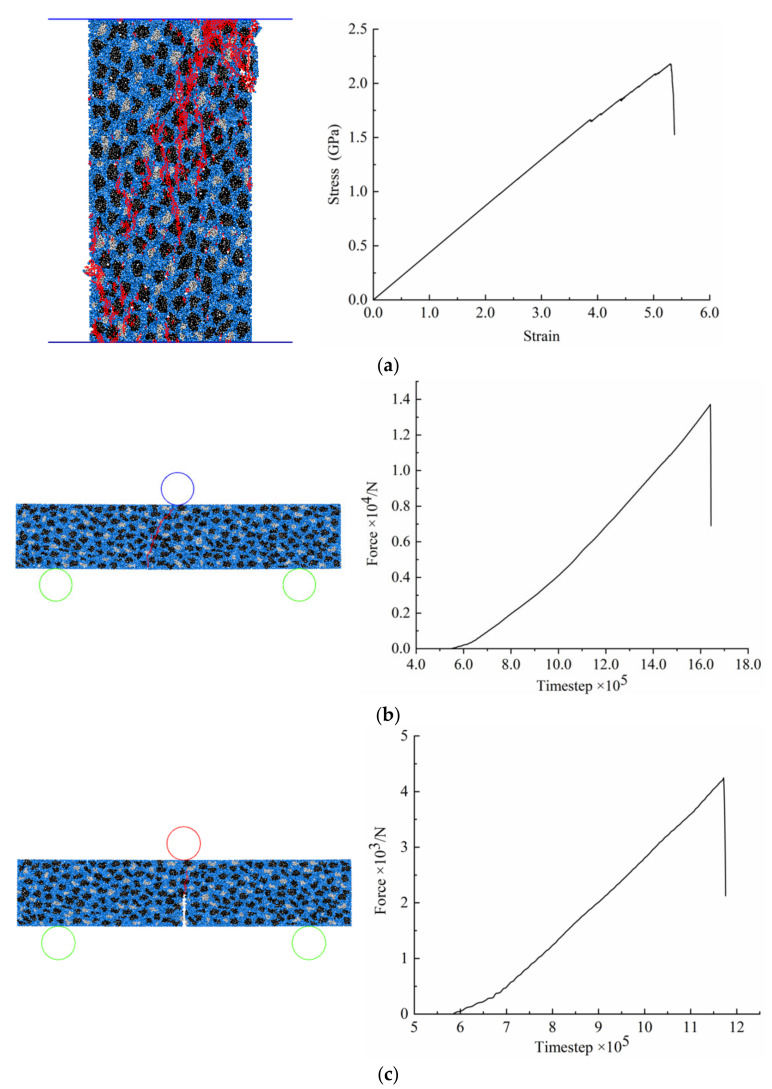
Numerical calibration tests: (**a**) compression test, (**b**) three-point bending test, and (**c**) single edge notched beam test (red lines represent cracks).

In general, calibration by numerical testing is a tedious trial-and-error process relying on the corresponding physical test results. In PFC2D, the compression test, three-point bending test and single-edge notch bending test were conducted to calibrate the Young’s modulus, Poisson’s ratio, bending strength and fracture toughness of the target solid specimen constructed above, as shown in Figure 4. According to the simulation results obtained from each test, these macroscopic properties of the target solid specimen could be calculated and then compared with the corresponding experimental results reported in [23]. It was found, as shown in Table 2, that the major macroscopic properties of the solid specimen agreed well with those of the real RB-SiC ceramic, so that it may be stated that this grain-based DEM model can be reasonably used to represent the mechanical behaviours of the RB-SiC ceramic in a material removal process.

## 3. Model of the Scratching of RB-SiC Ceramic

**Figure 5:**
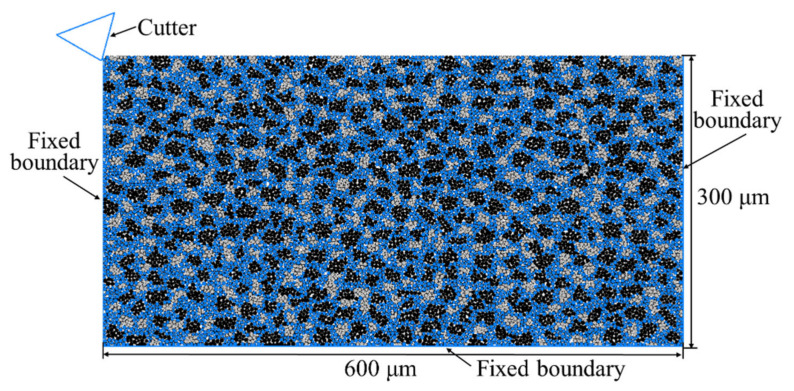
Model geometry and boundary conditions.

The geometry of the solid specimen, i.e., RB-SiC ceramic, was modelled as shown in Figure 5, where the top face of the specimen that would be scratched by a cutter was set as free, and the other three faces were set as fixed boundaries. In order to eliminate the specimen size effect on the simulation results, specimens with three different dimensional sizes were tested at a scratching speed of 20 m/s and cutting depth of 5 μm by using a rigid cutter with a cutting-edge radius of 1μm, a negative rake angle of 15° and a clearance angle of 30°, and then the total number of cracks and maximum depth of subsurface damage were measured, as given in Table 3. It was found that when the model dimensions were varied from 500 × 250 μm^2^ to 800 × 400 μm^2^, there was no obvious change in terms of the total number of cracks and the maximum depth of subsurface damage, such that the solid specimen with the dimension of 600 × 300 μm^2^, consisting of 13,079 spherical elements, was selected for the following simulations to achieve an accurate solution and reduce the computation time.

In simulation, four levels of scratching speed, *v_s_*, at 5, 10, 20 and 30 m/s, and depth of cut, *h_s_*, at 0.2, 0.4, 1 and 2 μm, were selected, and a full factorial experimental design was used for the scratching speed and depth of cut, which resulted in 16 combinations. Each of the 16 combinations was tested by using a rigid cutter with a negative rake angle of 15°, clearance angle of 30° and cutting-edge radius, *R*_0_, of 1 and 5 μm, respectively, so that a total of 32 tests were conducted with this grain-based DEM model for evaluating the surface integrity of scratching RB-SiC ceramic.

## 4. Results and Discussion

### 4.1. Overall Material Removal Mechanisms

**Figure 6:**
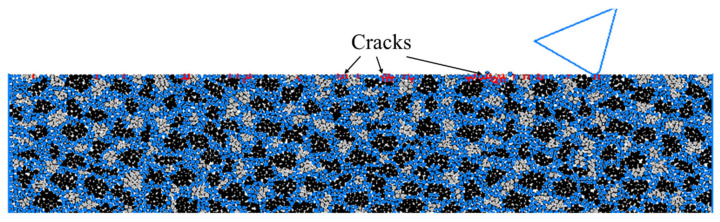
Surface/subsurface morphology after the scratching process in a ductile material removal mode (*v_s_* = 10 m/s, *h_s_* = 0.1 μm and *R*_0_ = 5 μm).

In general, there are three material removal processes in the scratching of hard brittle materials. When the depth of cut is small enough, only elastic-contact sliding occurs between the cutter and workpiece, such that there is no material removal from the target surface. When the depth of cut increases, a large scratching force generates in the cutter–workpiece contact zone, and the cutter may squeeze the workpiece to form a plastic deformation on both sides, where this type of plastic deformation results in ductile mode material removal. With a further increase in the depth of cut, the contact force between the cutter and workpiece is higher than the inherent contact force inside the workpiece, and hence, brittle fracture occurs to remove the material from the target. Thus, the transition of material removal mode from ductile to brittle fracture can be identified in the scratching of hard brittle materials, where the depth of cut presents the significant role in affecting this transition phenomenon. A previous study [24] indicates that the relationship between the material properties and the critical depth of cut is taken from:

(5) $$h_{c}=\psi \left(\frac{E}{H}\right){\left(\frac{K_{C}}{H}\right)}^{2}$$

where *E*, *K_C_*, *H* are the Young’s modulus, fracture toughness and hardness of the target material, respectively, and *ψ* is a constant which is usually valued at 0.15. By substituting the material properties of the RB-SiC ceramic, as given in Table 2, into Equation (5), the critical depth affecting the ductile–brittle transition of the RB-SiC ceramic in the material removal process was obtained, which was about 0.1 μm. As such, a scratching test on the modelled solid specimen was conducted with a scratching speed of 10 m/s and cutting depth of 0.1 μm by using a rigid cutter with a cutting-edge radius of 5 μm, a negative rake angle of 15° and clearance angle of 30°, and the surface/subsurface morphology is shown in Figure 6. It was found that only limited cracks (a total number of 46) were exposed around some spherical elements which were not detached from the target surface, and by repeating the scratching process with this critical depth of cut, these spherical elements with cracks around them would easily detach from the target surface, resulting in material removal without the formation of obvious median or lateral cracks in the target subsurface. Thus, it may be deduced that this is a ductile material removal mode in the scratching of RB-SiC ceramic.

**Figure 7:**
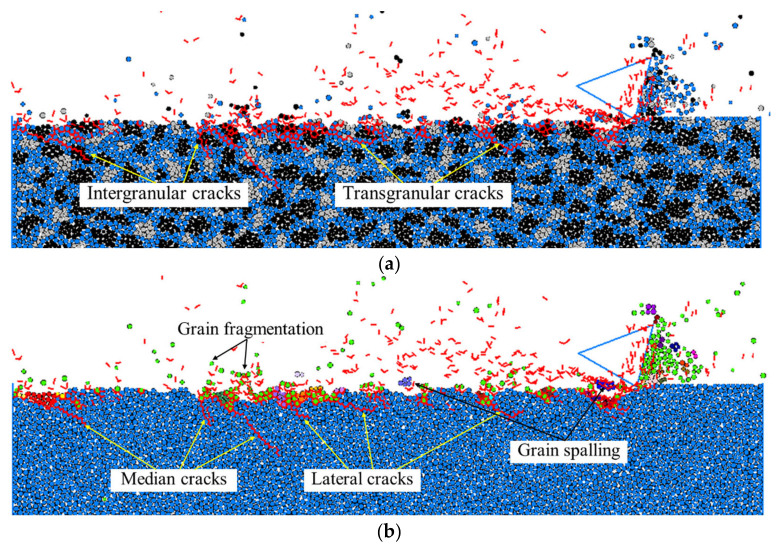
Surface/subsurface morphology after the scratching process in brittle material removal mode (*v_s_* = 20 m/s, *h_s_* = 1 μm and *R*_0_ = 1 μm). (**a**) Representation of transgranular and intergranular cracks. (**b**) Representation of median and lateral cracks (colour spherical elements, except blue, represent material removal in terms of grain fragmentation or grain spalling).

Moreover, the real brittle material removal mode in the scratching of RB-SiC ceramic is somewhat similar to that shown in Figure 7, where this test was conducted on the solid specimen with a scratching speed of 20 m/s and cutting depth of 1 μm by using the same cutter as above. It was found from the surface morphology that the detachment of spherical elements from the target was attributable to the initiation, propagation and intersection of cracks formed by the large contact force between the cutter and workpiece. To be specific, these cracks occurred both inside and along the cluster boundaries with the corresponding transgranular and intergranular cracks of RB-SiC ceramic, respectively, as shown in Figure 7a. It is also interesting to note from Figure 7b that the median cracks generally propagated perpendicularly, deep into the target, while some of the lateral cracks propagated parallel to the target surface, and some propagated to the target surface and interacted with other surface cracks resulting in material removal from the workpiece. To be specific, the green spherical elements represent material removal in terms of grain fragmentation, which is mainly caused by intergranular cracks, while the spherical elements of other colours, except blue, represent material removal in terms of grain spalling that is primarily caused by transgranular cracks. Therefore, the grain-based DEM model can be properly employed to represent the scratching of RB-SiC ceramic by identifying the different material removal mechanisms, such as the ductile and brittle material removal modes, and it can also be used to observe the dynamic initiation, propagation and, eventually, interaction of cracks occurring both inside and along the grain boundaries, which is hard to observe by experiments, such that numerical simulation with the grain-based DEM model is an effective and viable alternative method for evaluating the surface integrity in the scratching of RB-SiC ceramic.

### 4.2. Effect of Processing Parameters on the Subsurface Integrity

#### 4.2.1. Scratching Speed

**Figure 8:**
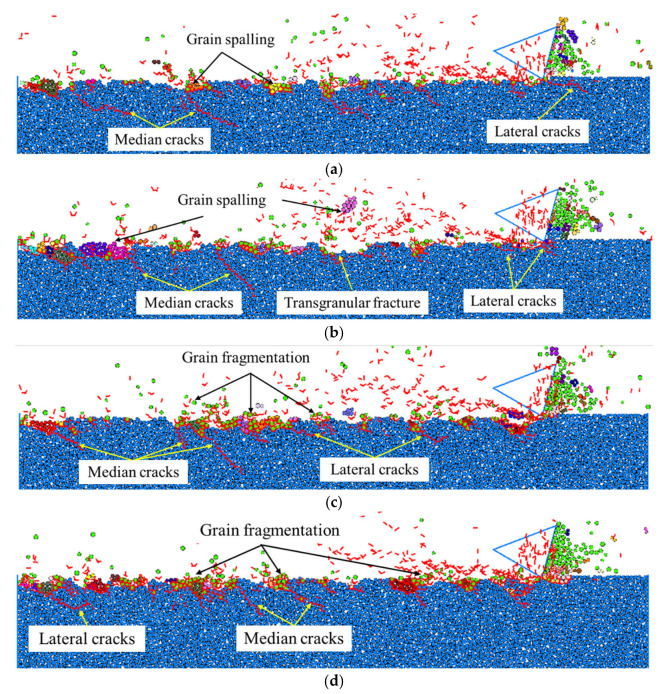
Surface/subsurface morphology after the scratching process under different scratching speeds (*h_s_* = 1 μm and *R*_0_ = 1 μm): (**a**). *v_s_* = 5 m/s, (**b**). *v_s_* = 10 m/s, (**c**). *v_s_* = 20 m/s, and (**d**). *v_s_* = 30 m/s.

It can be seen from Figure 8, that with the same depth of cut and cutting-edge radius of cutter, the surface/subsurface morphologies under different scratching speeds were significantly different. As can be observed from Figure 8a, when the scratching speed was 5 m/s, a large number of median cracks propagated to the target subsurface, while most of the lateral cracks propagated along the grain boundaries to the target surface, resulting in large grain spalling from the target surface, and leaving a relatively rough scratching surface. With the increase in the scratching speed to 10 m/s, the remaining large grain spalling dominated the material removal process, which was mainly caused by the interaction of lateral cracks, leading to typical transgranular fracture on the target surface, as shown in Figure 8b. However, with the further increase in the scratching speed to 20 m/s, as noted in Figure 8c, some of the lateral cracks could not propagate to the target surface but propagated parallel to the target surface, which did not cause material removal from the target. In this situation, the intergranular cracks propagating through the grains dominated the material removal in terms of grain fragmentation with a small amount of small grain spalling, by which a relatively smooth scratching surface was obtained. When the scratching speed increased to 30 m/s, the resultant grain fragmentation caused by the intergranular cracks with the small amount of small grain spalling still dominated material removal from the target surface, as can be seen from Figure 8d; in particular, the material removed in front of the cutter rake face was mainly by grain fragmentation. However, it was also interesting to notice that the overall length of median cracks on the target subsurface significantly decreased.

**Figure 9:**
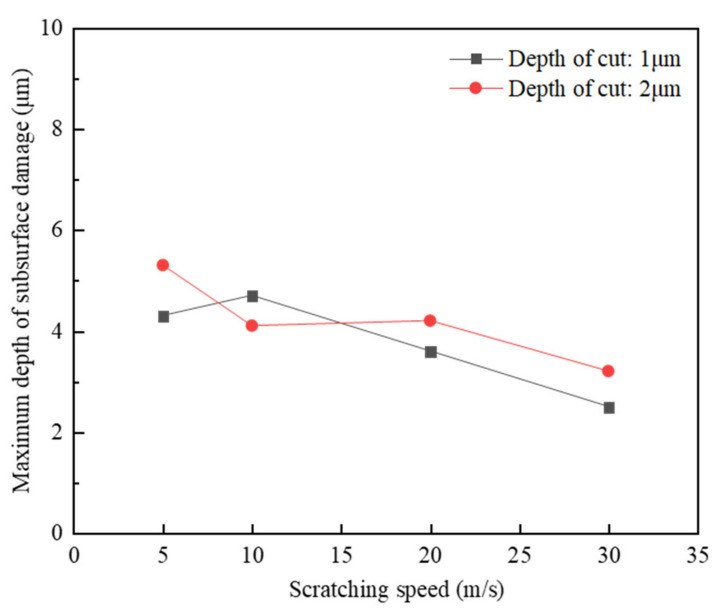
Effect of the scratching speed on the maximum depth of subsurface damage.

A further quantitative study was conducted to assess the relation between the scratching speed and the maximum length of the median cracks in the perpendicular direction of the target surface, which was regarded as the maximum depth of subsurface damage. The quantitative comparison in Figure 9 shows that with the increase in scratching speed, the maximum depth of the subsurface damage decreased. As the scratching force is relatively large at the low scratching speed that facilitates the occurrence of the transgranular cracks, there was large grain spalling from the target surface and the propagation of median cracks deep into the target subsurface. In contrast, the scratching force is relatively small at high scratching speed, and could cause the primary material removal in terms of grain fragmentation due to the intergranular cracks, which can prohibit the propagation of cracks to the depth of the target subsurface [25]. Therefore, to increase the scratching speed, a good surface integrity is required.

#### 4.2.2. Depth of Cut

**Figure 10:**
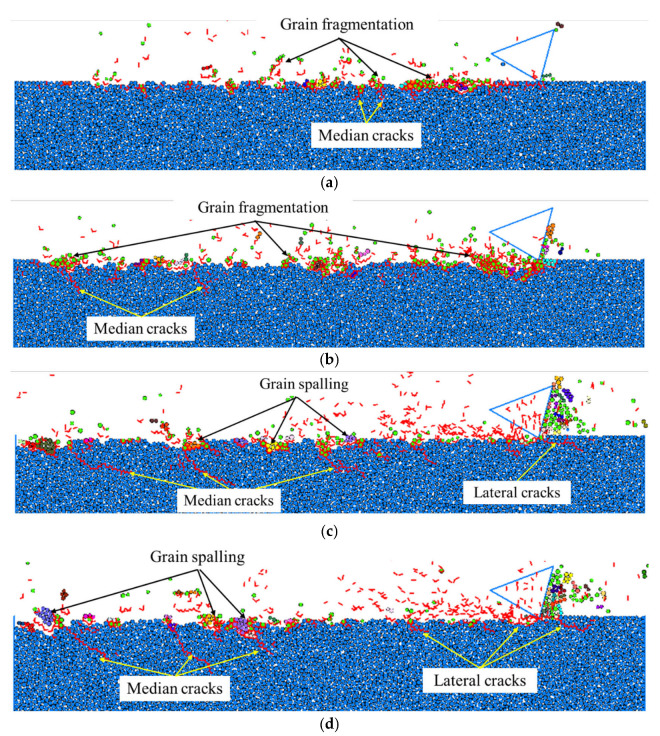
Surface/subsurface morphology after the scratching process under different depths of cut (*v_s_* = 5 m/s and *R*_0_ = 1 μm): (**a**). *h_s_* = 0.2 μm, (**b**). *h_s_* = 0.4 μm, (**c**). *h_s_* = 1 μm, and (**d**). *h_s_* = 2 μm.

The effect of depth of cut on surface integrity, on the scratching of a solid specimen, i.e., RB-SiC ceramic, was explored under the same scratching speed of 5 m/s and cutting-edge radius of 1 μm. It can be seen from Figure 10a, that when the depth of cut was 0.2 μm, it was just larger than the critical depth of cut, 0.1 μm, for the occurrence of ductile–brittle transition in the scratching of RB-SiC ceramic. On the target surface, material removal was mainly dominated by grain fragmentation caused by the intergranular cracks with limited small grain spalling, while on the target subsurface it was found that only a few median cracks propagated deep into the target with small lengths. By increasing the depth of cut to 0.4 μm, obvious median cracks were seen, see Figure 10b, propagating to the target subsurface with relatively long lengths, and both grain fragmentation and grain spalling were found before and behind the cutter on the target surface. Then, many occurrences of large grain spalling caused by transgranular cracks were found on the scratched target surface by further increasing the depth of cut to 1 μm, thus, resulting in typical brittle material removal with large fractures. It was also noted that in this situation the subsurface damage consisted of obvious median cracks and lateral cracks. Similar material removal mechanisms can be found in Figure 10d, where the depth of cut was increased to 2 μm, and the maximum depth of subsurface damage continued to propagate deep into the target subsurface in a perpendicular direction.

**Figure 11:**
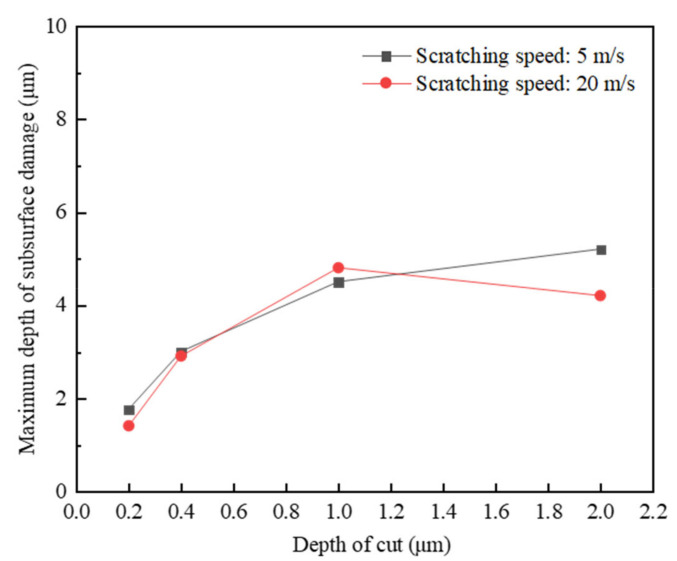
Effect of the depth of cut on the maximum depth of subsurface damage.

Similarly, a quantitative comparison was also carried out to evaluate the relation between the depth of cut and the maximum depth of subsurface damage, and the result is shown in Figure 11, where the maximum depth of subsurface damage shows an increasing trend with the increase in the depth of cut. This was attributed to the fact that the scratching force increases by increasing the depth of cut [26], and when the depth of cut is small, the scratching force induces intergranular cracks, primarily dominating material removal in terms of grain fragmentation, and, thus, resulting in the relatively smooth scratched target surface, also with limited subsurface damage. When the depth of cut is large enough, the scratching force induces transgranular cracks, and lateral cracks interact with each other, causing the large grain spalling to detach from the target surface and subsurface damage with long median cracks. Consequently, in order to obtain a better surface finish and reduce the subsurface damage during high-speed abrasive machining processes, such as grinding and polishing processes, it is recommended to reduce the depth of cut if the material removal rate is not a primary concern.

#### 4.2.3. Cutting-Edge Radius

**Figure 12:**
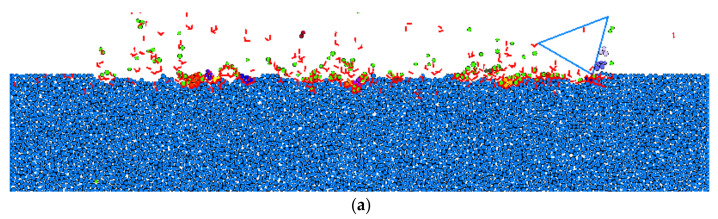

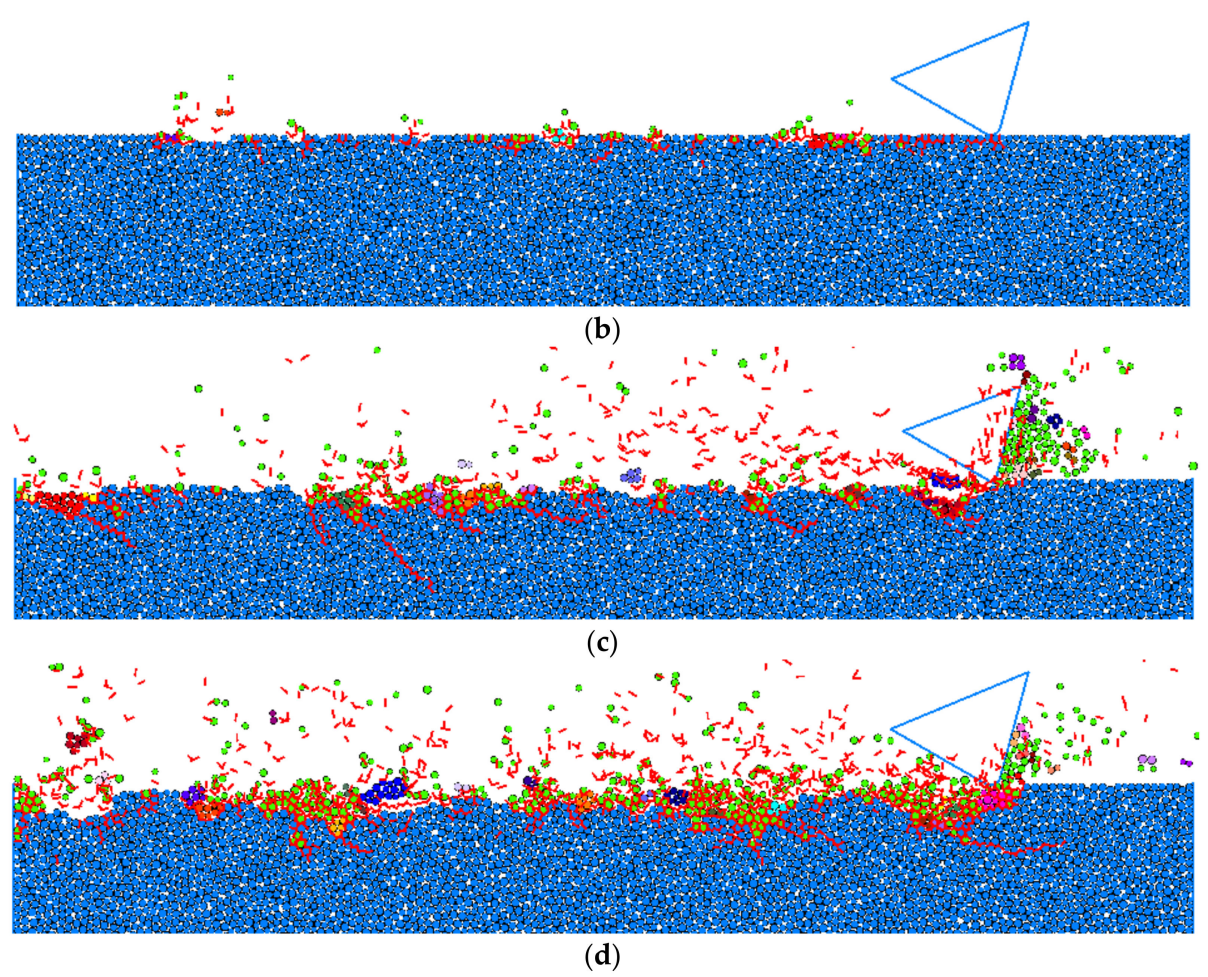
Surface/subsurface morphology after the scratching process under different cutting-edge radii of the cutter: (**a**) *v_s_* = 20 m/s, *h_s_* = 0.2 μm and *R*_0_ = 1 μm; (**b**) *v_s_* = 20 m/s, *h_s_* = 0.2 μm and *R*_0_ = 5 μm; (**c**) *v_s_* = 20 m/s, *h_s_* = 1 μm and *R*_0_ = 1 μm; (**d**) *v_s_* = 20 m/s, *h_s_* = 1 μm and *R*_0_ = 5 μm.

For metal cutting, employing a relatively smaller cutting-edge radius can facilitate the micro-cutting action of the cutter to obtain a better surface finish with lower roughness [27]. However, for the cutting of hard brittle materials such as ceramics, the effect of the cutting-edge radius on surface integrity seems to be different. Due to the stress concentration in the cutter–workpiece contact zone, by using a cutter with a cutting-edge radius of 1 μm on the target surface, both grain fragmentation and grain spalling, as observed in Figure 12a, and limited residual cracks, were also found on the target subsurface. In contrast, only a small amount of material removal from the solid specimen in terms of grain fragmentation was found on the target surface by using a cutter with cutting-edge radius of 5 μm, and, thus, presenting a good subsurface quality as shown in Figure 12b, by which it may be deduced that increasing the cutting-edge radius of the cutter would enhance the ductile machinability of the target. On increasing the depth of cut to 1 μm, although the subsurface damage seemed to be better after the scratching process with a cutting-edge radius of 5 μm, than with a cutting-edge radius of 1 μm, which consisted of many large median and lateral cracks, as seen by comparing Figure 12c,d, the surface quality seemed to be worse, with large grain spalling detaching from the target surface due to the increased scratching force which occurred by employing the relatively big cutting-edge radius.

## 5. Conclusions

A novel grain-based DEM model for representing RB-SiC ceramic and its associated scratching process has been developed in this paper to explore the material removal mechanisms and surface integrity induced by scratching. A combination of the cluster and parallel bond models was used to construct the target solid specimen, i.e., RB-SiC ceramic, by considering the bonded SiC and Si grains and cementitious materials in PFC2D. The macroscopic properties of the constructed target solid specimen were then verified by material numerical tests, namely, compression test, three-point bending test and single-edge notch bending test. It was shown that the macroscopic properties of the solid specimen in the developed grain-based DEM model agreed well with those of the real target material, RB-SiC ceramic.

The model was then used to simulate, and give an insight into, the scratching process under various processing parameters. It was found that the grain-based DEM model can properly and accurately represent the material removal process by identifying the transgranular and intergranular cracks, the ductile erosion, and the brittle material removal mode, in terms of grain fragmentation and grain spalling. It was further found that increasing the scratching speed or decreasing the depth of cut can result in a decrease in the maximum depth of subsurface damage. This is because the scratching force is relatively large at the low scratching speed, or because the large depth of cut facilitates the occurrence of transgranular cracks, the large grain spalls from the target surface and the median cracks are propagated deep into the target subsurface. Furthermore, the effect of the cutting-edge radius on surface integrity, on scratching of the solid specimen, was also investigated. It was shown that increasing the cutting-edge radius of the cutter enhanced the ductile machinability of the target and reduced the target subsurface damage. This proposed novel grain-based DEM model provides an alternative avenue to effectively evaluate surface integrity in the machining of ceramics, and by employing this model, the effect of the geometry of the cutting edge on the surface integrity will be further explored in future work.

## Figures and Tables

**Table 1:** Input microscopic properties for the grain-based DEM model.

Classifications	Microscopic Properties	Values
Basic spherical element	Minimum spherical element size in radius (μm)	1.5
	Maximum spherical element size in radius (μm)	2.4
	Density of the spherical elements (kg/m^3^)	3100
	Friction factor between spherical elements	0.7
Cluster: cementitious materials	Young’s modulus (GPa)	235
	Ratio of shear to normal stiffness	1.33
	Tensile strength (MPa)	620
	Shear strength (MPa)	4340
Cluster: SiC grains	Young’s modulus (GPa)	245
	Ratio of shear to normal stiffness	1.33
	Tensile strength (MPa)	1150
	Shear strength (MPa)	7950
Cluster: Si grains	Young’s modulus (GPa)	245
	Ratio of shear to normal stiffness	1.33
	Tensile strength (MPa)	950
	Shear strength (MPa)	6650

**Table 2:** Comparison of macroscopic properties between real RB-SiC ceramic and grain-based DEM modelling solid specimen.

Macroscopic Properties	RB-SiC Ceramic [23]	Grain-Based DEM Modelling Solid Specimen	Errors (%)
Young’s modulus (GPa)	430	435	1.2
Bending strength (MPa)	490	482.4	1.6
Poisson’s ratio	0.16	0.159	0.63
Fracture toughness (MPa·m^0.5^)	3.5	3.4	2.9

**Table 3:** Effect of specimen size on simulation results.

Specimen Size (μm^2^)	Number of Spherical Elements	Total Number of Cracks	Maximum Depth of Subsurface Damage (μm)
500 × 250	9085	1023	2.5
600 × 300	13,079	1031	2.4
800 × 400	23,103	1047	2.3
